# Quantitative genetic analyses provide parameters for selection and conservation of captive Great-billed Seed-finches (*Sporophila maximiliani*)

**DOI:** 10.1371/journal.pone.0236647

**Published:** 2020-07-30

**Authors:** Mário L. Santana

**Affiliations:** Grupo de Melhoramento Animal de Mato Grosso (GMAT), Instituto de Ciências Agrárias e Tecnológicas, Universidade Federal de Rondonópolis, Rondonópolis, Mato Grosso, Brazil; University of Missouri Columbia, UNITED STATES

## Abstract

The Great-billed Seed-finch (*Sporophila maximiliani*) is an endangered South American bird that has suffered from trafficking and the destruction of its natural habitat. In contrast, there are over 180,000 Great-billed Seed-finches legally raised in captivity in Brazil. The interest as a pet for Great-billed Seed-finches is due to their exceptional ability to sing. In the present research, the unknown genetic structure of the Great-billed Seed-finch captive population was investigated by quantitative analysis of 6,226 pedigree records. Additionally, 7,671 phenotypic records were available to estimate genetic parameters such as heritability and evolvability of a song-related trait of these birds for competitions. The captive Great-billed Seed-Finch population faces many of the problems commonly encountered in domestic animal populations such as a high level of inbreeding (average of 8.26%, 70.47% of birds were inbred), pedigree bottlenecks, unbalanced contribution of breeding animals and structuring (equivalent number of subpopulations of 2.91). Despite this, most genetic diversity remains preserved within aviaries. The high generation interval (5.74 years) found for this population should help to prevent a rapid increase in inbreeding and genetic drift. These results should serve as strong motivation and support for urgent actions to manage the genetic diversity of captive Great-billed Seed-Finches. From the viewpoint of genetic improvement for singing time in tournaments (STT), this trait presents sufficient variability to allow response to artificial selection given the heritability of 18.7% and evolvability of 2,447%. In contrast, inbreeding and high generation interval appear to be the most considerable barriers that may limit the genetic gain for STT. Widespread adoption of techniques such as optimal genetic contribution selection and implementation of routine genetic diversity monitoring via pedigree analysis and molecular tools can be crucial both in terms of breeding and conservation of genetic diversity of captive Great-billed Seed-Finches.

## Introduction

The Great-billed Seed-finch (*Sporophila maximiliani*) is a South American bird, occurring mainly in flooded areas and marshy borders in the Brazilian Cerrado biome [1]. This little bird has suffered from trafficking and the destruction of its natural habitat. Currently, Great-billed Seed-finch is rarely found in nature [1] and is therefore regarded as endangered by the International Union for Conservation [2] and as critically endangered by the Brazilian government (ICMBio-MMA, 2018). Probably due to the small number of free individuals, records of trafficking of Great-billed Seed-finches today have been relatively rare [3–6]. In contrast, there are over 180,000 Great-billed Seed-finches legally raised in captivity in Brazil [4,7].

The interest as a pet for Great-billed Seed-finches is due to the characteristics related to the quality of their singing, diversity of melodies, beauty, territorial behavior, ease of management, and adaptation to the domestic environment. For decades, the characteristics of Great-billed Seed-finches have stimulated the holding of tournaments of the modalities called song quality and fiber in Brazil. These competitions are organized by breeders associations such as the Brazilian Confederation of Native Bird Breeders (COBRAP) with the proper permission of local environmental authorities. Events take place regularly in various regions of Brazil, and some have gathered up to 400 Great-billed Seed-finches. In song quality tournaments, birds are kept isolated from others of the same species and judged individually for various attributes related to the quality and complexity of their song. In fiber tournaments, birds kept collectively under visual contact are evaluated for the total time they sang within 15 minutes. Fiber tournaments are currently of greater interest to breeders and stimulate the economy of many Brazilian cities, whether through the legal trade of the birds themselves as well as accessories, food, and drugs. Commercial interest has increased and contributed to a great appreciation for Great-billed Seed-finches. In popular commercial aviaries, the average price for a young bird is usually between 800 and 8,000 American dollars. This value can increase considerably depending on gender, age, and especially the achievement of tournaments by the individual himself or his ancestors.

The high interest of breeders in birds that perform well in fiber competitions has directly influenced the selection decisions and mating practices of captive Great-billed Seed-finches. Often, matings are performed among genetically related individuals, which, together with the unbalanced use of breeding animals, can contribute to the increase of inbreeding and loss of genetic diversity. Also, there is an interest of breeders to preserve their pure lineages selected for song quality or fiber, avoiding genetic exchange between aviaries. This practice could lead to the subdivision of the captive population. On the one hand, the subdivision of a population could contribute to minimizing the risk of extinction due to accidental or sanitary causes, since the occurrence of these events would affect only one group. On the other hand, each subpopulation would have a relatively low effective population size and a higher level of inbreeding [8]. The increase in inbreeding in domestic animal populations has been widely associated with damage to traits of economic interest [9]. In birds, for example, the inbreeding affected the song phonetics [10], song rate, beak color, fat deposition, body size, male attractiveness to females, and the female choice behavior [11]. Inbreeding also led to the early death of individuals [12], reduced incubation attentiveness and hatching success [13], lower sperm motility, and a higher percentage of abnormal spermatozoa in the ejaculate [14]. Unfortunately, until today, almost nothing is known about the population structure and genetic diversity of Great-billed Seed-finches captives. Knowledge and monitoring of parameters related to inbreeding, structure, and genetic diversity of the population is important for the conservation of captive Great-billed Seed-finches and the viability of reintroduction projects such as that proposed by [7].

The song of birds is closely related to recognition, sexual selection, and male-male competition [15–17]. Therefore, it is great the interest of Great-billed Seed-finch breeders by the selection for traits related to singing and territorialism. However, there is no knowledge about the capacity of the captive population of Great-billed Seed-finches to respond to artificial selection. Heritability, as well as evolvability of a trait, is a parameter used to measure evolutionary potential at the population level [18,19]. In the narrow sense, heritability expresses the extent to which phenotypes are determined by the genes transmitted by the parents [20]. However, for purposes of comparing the expected response potential of different traits, evolvability can be considered a more appropriate measure [19]. Despite the importance of these parameters for conservation and selection purposes, estimates of heritability and evolvability for bird song-related traits are relatively scarce [21,22]. One of the main limitations for obtaining these parameters is the difficulty in obtaining pedigree information from captive birds and particularly free-living birds [23,24], as well as quantity and quality phenotypes. The estimation of genetic parameters for traits of selection interest, such as Great-billed Seed-finches song, would enable the prediction of breeding values [18]. Also, the availability of genetic parameters enables the implementation of tools to monitor and control the inbreeding rate while maximizing genetic gain [25].

In the present research, the unknown genetic structure of the Great-billed Seed-finch captive population was investigated by quantitative analysis of pedigree records. Inbreeding level, parameters related to genetic diversity, structuring, generation interval, and others were revealed. In a second step of the research, phenotypic and pedigree records were used together to estimate parameters such as heritability and evolvability of a song-related trait. Finally, the value and possible applications of the results achieved for the selection and conservation of captive Great-billed Seed-finches were discussed.

## Materials and methods

### General data information

Pedigree and phenotypic records of captive Great-billed Seed-finches were obtained from a pre-existing database maintained by the extension project entitled "GEMAXI—Genetic Evaluation of Native Passerines for Traits of Interest" (Federal University of Mato Grosso, Federal University of Rondonópolis, Brazil (SIEX-CAMEX-PROCEV: 260220181145431760). The database used is a sample that represents well the current population of captive Great-billed Seed-finch, as it comprises information from most popular Brazilian aviaries. Pedigree information comprised up to 13 generations of birds born between December 1989 and May 2019, totaling 6,226 birds. The complete phenotype database consisted of 7,671 records obtained from 625 birds between 2005 and 2019.

### Pedigree analysis

All parameters were computed for the reference population of birds born from 2008 to 2019. This definition is equivalent to birds born in the last two generation intervals, that is, representing the current population of Great-billed Seed-finch. The generation interval was defined as the average age of parents at the birth of their progeny kept for reproduction. Pedigree completeness was accessed by the number of equivalent complete generations (ECG) traced, computed as the sum over all known ancestors of the terms (1/2^*d*^), where *d* is the ancestor's generation number, which is equal to one for the parents, two for the grandparents, etc. [26].

The F-statistics [27] based on pedigree information were computed to characterize the genetic structure of the Great-billed Seed-finch population. The average inbreeding coefficient of birds was denominated *F*_*IT*_, and the average inbreeding coefficient under random mating was *F*_*ST*_. The *F*_*ST*_ was computed from a hypothetical population produced by matching males and females randomly. The statistic *F*_*IS*_ was computed as:

$$F_{IS}=\frac{F_{IT}-F_{ST}}{1-F_{ST}}.$$

Effective population sizes were estimated based on individual rates of inbreeding Δ*F*_*i*_ [28,29] and coancestry Δ*C*_*ij*_ [30], considering: ${\Delta F}_{i}=1-\sqrt[{t_{i}-1}]{\left(1-F_{i}\right)}$ and ${\Delta C}_{ij}=1-\sqrt[{(t_{i}+t_{j)/2}}]{\left(1-C_{ij}\right)}$, where *F*_*i*_ is the inbreeding coefficient of the individual *i*, *C*_*ij*_ is the coancestry coefficient between individuals *i* and *j*, and *t*_*i*_ and *t*_*j*_ are their respective ECG. Finally, the following formulas were used: ${NeF}_{i}=1/(2\bar{\Delta F})$ and ${NeC}_{i}=1/(2\bar{\Delta C})$. The number of equivalent subpopulations was computed as suggested by [30,31]: *S* = *NeC*_*i*_/*NeF*_*i*_.

The main ancestors (founders or not) of the reference population were identified as described in [32]. The marginal contribution of each main ancestor was computed as its expected genetic contribution not yet explained by the other ancestors. The genetic contribution of a founder was calculated as the probability that a gene randomly taken within the reference population came from the founder [33]. The effective number of founders (*f*_*e*_) and ancestors (*f*_*a*_) was obtained to evaluate the concentration of the origin of both animals and genes. Parameter *f*_*e*_ is defined as the number of equally contributing founders that would be expected to produce the same genetic diversity as observed in the population under study [34]. This parameter was calculated as $f_{e}=1/(\sum_{k=1}^{f}q_{k}^{2})$, where *q*_*k*_ is the probability of gene origin of founder *k*. Parameter *f*_*a*_ is the minimum number of ancestors, not necessarily founders, explaining the complete genetic diversity of a population. This parameter was calculated as $f_{a}=1/(\sum_{j=1}^{a}q_{j}^{2})$, where *q*_*j*_ is the marginal contribution of ancestor *j*, which is the genetic contribution made by an ancestor that is not explained by other ancestors chosen before. The founder genome equivalents (*f*_*g*_) can be defined as the number of founders that would be expected to produce the same genetic diversity as observed in the population under study if the founders were equally represented and no loss of alleles occurred [34]. Following [35], the parameter *f*_*g*_ was obtained by the inverse of twice the average coancestry of the individuals included in a pre-defined reference population.

The genetic diversity (GD) in the reference population was computed as $GD=1-\frac{1}{2f_{g}}$ [34,36]. In both natural and domestic populations, bottlenecks and genetic drift occur frequently and lead to loss of genetic diversity. The genetic diversity loss in the population since the founder generation can be estimated by 1–*GD*. The loss of genetic diversity due to unequal contributions of founders was estimated by 1–GD*, where ${GD}^{*}=1-\frac{1}{2f_{e}}$ [35]. The difference between *GD* and GD* indicates the genetic diversity loss due to genetic drift that had accumulated since the founding of the population [36]. Thus, this difference can be expressed as $GD-{GD}^{*}=\frac{1}{2f_{n}}$, where *f*_*n*_ is the effective number of non-founders. This parameter was computed by the following expression proposed by [35]: $\frac{1}{f_{g}}=\frac{1}{f_{e}}+\frac{1}{f_{n}}$.

Within the reference population, the birds of ten of the most popular commercial aviaries in Brazil were identified. For these aviaries was calculated the Nei's minimum genetic distance [37] as *D*_*ij*_ = [(*C*_*ii*_+*C*_*jj*_)/2]−*C*_*ij*_, where *C*_*ij*_ is the average pairwise coancestry between birds of aviaries *i* and *j*, including all *N*_*i*_×*N*_*j*_ pairs, and *C*_*ii*_ and *C*_*jj*_ are the average pairwise coancestries within aviaries *i* and *j*, respectively. A distance matrix (10 × 10) was constructed, which has 0 on the diagonals and *D*_*ij*_ on the off-diagonals. A cluster analysis was applied to the distance matrix using the unweighted pair group method with arithmetic mean and a dendrogram was produced.

The pedigree analyses were performed using the PEDIG [38] and RELAX2 [39] software and cluster analysis using the R cluster function [40].

#### Trait definition and phenotypic records

**Fig 1:**
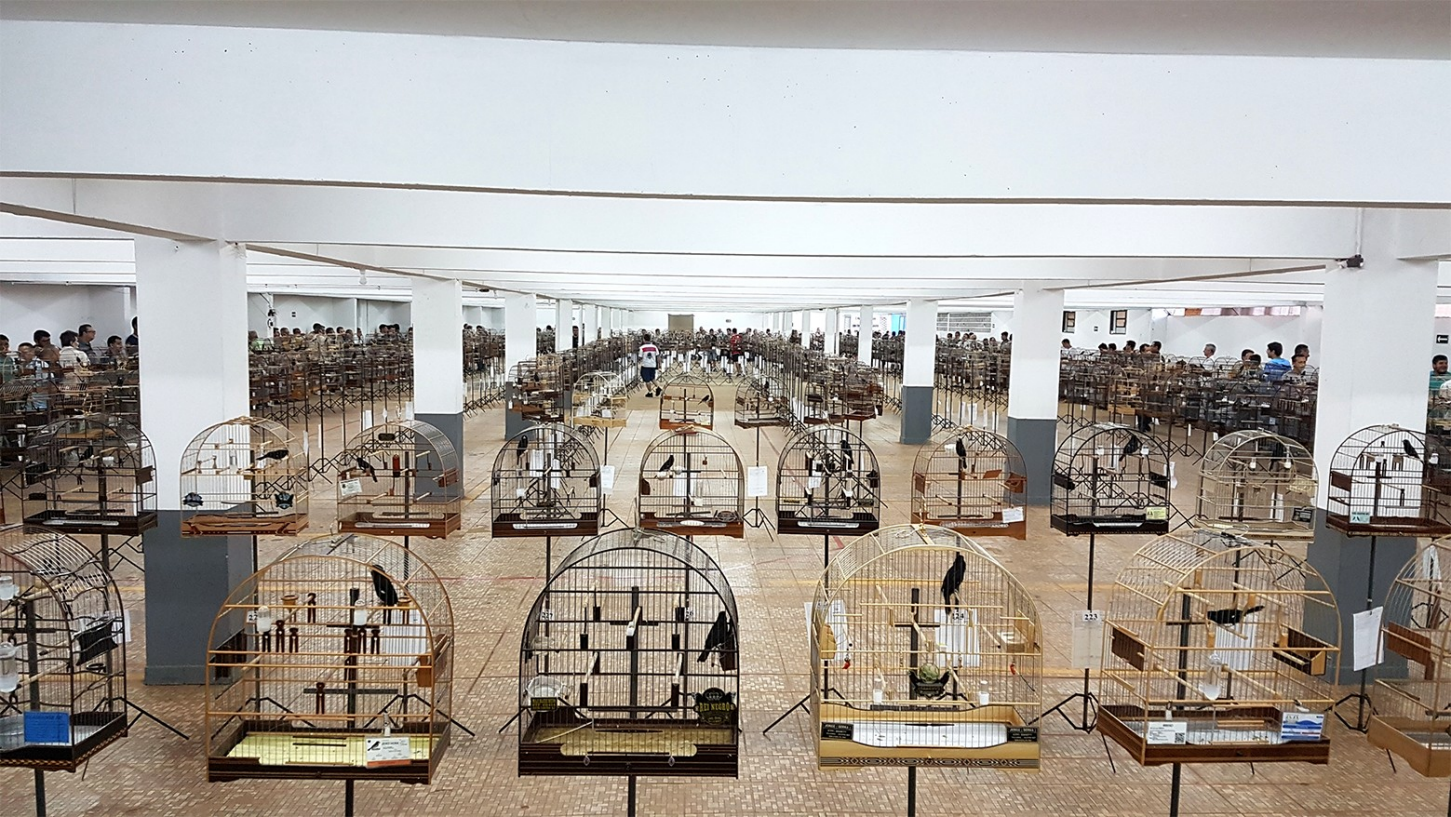
A typical national Great-billed Seed-finch competition, fiber modality. More than 300 birds participated in this event.

Fiber tournaments are very popular for several bird species in Brazil, each with its own rules. For Great-billed Seed-finches, the competitions were held in large gyms with good lighting and ventilation (Fig 1). The birds were individually housed in cages (length 50, depth 22, height 53 cm, approximately), which were fixed to 1.5 m high metal stakes. The stakes were arranged in a circular shape with a distance of 20 cm between each cage. The visual contact among the birds was allowed. When large numbers of birds participated in a single event, additional circles of cages were formed within the main circle (Fig 1). All birds had *ad libitum* access to water, food, and a bathtub. Almost all birds participating in the competitions had a female with which they were mated. The mating of Great-billed Seed-finches is a crucial point in stimulating male-male competition. In the reproductive period, these birds become extremely territorial and aggressive [1]. The female of each male, also individually caged, accompanied his mate until the tournament began. From this moment on, the females were moved to a place with sound and visual isolation of the males. The events were held in several cities in the southern, southeastern, and midwestern regions of Brazil, beginning at 08:00 am and lasting approximately 4 hours. A director-general, inspectors, and staff trained to perform the phenotypic recording of bird song performance formed the teams that organized and conducted the competitions. Each event has always been accompanied by a veterinarian to ensure the health and welfare of all participating birds. All events occurred with express authorization and supervision by local environmental authorities.

The trait analyzed in the present study was defined as singing time in tournaments (STT), measured in seconds. At around 12:00 pm, an electronic record of the total time of each bird sang within 15 minutes was recorded. Each technician positioned about 1.5 m in front of a bird's cage used a timer provided by the event organizer for the registration of the STT. The winning bird of an event was the one with the highest STT record.

### Estimation of genetic parameters for singing time in tournaments

Only birds whose pedigree were known as well as their hatch date were considered in this study. Contemporary groups were defined by birds that participated in the same tournament, i.e., in the same city, place, year, month, and day. Records of birds in contemporary groups with fewer than three animals were eliminated, as well as data exceeding 3.5 standard deviations above or below the mean of the trait within the respective contemporary group. Thus, after quality control of data, 7,549 records of 602 birds were effectively used to estimate genetic parameters. The pedigree file of birds with phenotypic records comprised 2,138 animals. A summary of the data set is shown in Table 1.

**Table 1:** Summary of data structure (standard deviation in parentheses) used to estimate genetic parameters for the singing time in tournaments (STT) of captive Great-billed Seed-Finches.

Item	Statistics
Birds in the pedigree, n	2,138
Males with progeny record, n	217
Females with progeny record, n	337
Birds with records, n	602
Records, n	7,549
Mean number of records per bird	12.54
Birds with ≤ 5 records, %	45.68
Birds with > 5 and ≤ 10 records, %	16.94
Birds with > 10 and ≤ 15 records, %	10.80
Birds with > 15 and ≤ 20 records, %	6.81
Birds with > 20 and ≤ 25 records, %	6.15
Birds with > 25 and ≤ 30 records, %	3.82
Birds with > 30 records, %	9.80
Mean of STT, seconds	321.35 (124.10)
Median of STT, seconds	324
Minimum STT, seconds	0
Maximum STT, seconds	759
Contemporary groups, n	730
Breeders, n	219
Mean age of bird at the tournament, years	5.73 (2.91)
Minimum age of bird at the tournament, years	1.48
Maximum age of bird at the tournament, years	18.75

The mixed model adopted to estimate the genetic parameters of STT included the fixed effects of contemporary group (as defined above), the age of the bird at the date of competition (linear and quadratic effects) and the random effects of animal (additive genetic and permanent environmental), breeder and residual. The (co)variance structure follows:

$$var\left[\begin{array}{c} a\\ c\\ b\\ e \end{array}\right]=\left[\begin{array}{cccc} A\sigma_{a}^{2} & 0 & 0 & 0\\ 0 & I\sigma_{c}^{2} & 0 & 0\\ 0 & 0 & I\sigma_{b}^{2} & 0\\ 0 & 0 & 0 & I\sigma_{e}^{2} \end{array}\right],$$

where *σ*^2^ are variances the additive genetic (*a*), permanent environmental (*c*), breeder (*b*), and residual (*e*) effects, respectively; *A* is the numerator of the relationship matrix among all birds, and *I* is the identity matrix. The variance components were obtained under a single trait repeatability animal model [41] using Gibbs sampling. All analyses were performed with the GIBBS2F90 program [42]. The prior distributions were noninformative inverse Wishart distributions for all random effects. The analysis consisted of a single chain of 650,000 cycles, with a conservative burn-in period of 150,000 cycles and a thinning interval of 50 cycles. Thus, 10,000 samples were effectively used for final inferences. Convergence was determined by graphical inspection of the posterior chains of the parameters using the program POSTGIBBSF90 [42]. Narrow-sense heritability and repeatability were calculated in the usual way [20]. Permanent environmental and breeder effects were expressed as the proportion of the phenotypic variance. Evolvability was calculated by dividing the additive genetic variance by the mean square of STT [19,43]. The percentage change in STT over *t* generations with evolvability *I*_*A*_ and strength of selection *β*_*μ*_ was computed as described in [19]: (1+*I*_*A*_*β*_*μ*_)^*t*^. The parameter *β*_*μ*_ was considered equal to 1, which means that a 1% change in the trait produces a 1% change in fitness.

## Results

### Pedigree analysis

#### Demographic parameters and pedigree completeness

Pedigree records of 6,226 birds from 925 males and 1,632 females were available. For 908 birds, one or both parents were not known. Of the total birds in the pedigree file, 2,538 (40.76%) were male and 3,688 (59.24%) female. The ratio of males to females in reproduction was approximately 1 to 1.8. This number reflected the adoption of polygamy as a mating practice. The average number of progeny was 5.95 and 3.28, while the maximum was 185 and 40 per father and mother, respectively. The average number of hatchlings per clutch was 1.47 (standard deviation of 0.59). Approximately 79% of hatches occurred between November and March, with 19.7% in January.

As previously reported, the reference population was defined based on the generation interval (Table 2) to reflect the active Great-billed Seed-Finch population. For this group of individuals, the parameters of the pedigree analysis were calculated. The overall average of the generation interval, considering 1,294 information, was 5.74 years. On average, the generation intervals were shorter for females and longer for males, probably due to the reproductive life of males being longer than females. In captivity, these birds can be very long-lived, which can be verified by the minimum (about ten months) and maximum (about 23 years) values of the generation interval.

**Table 2:** Generation interval (in years) of captive Great-billed Seed-Finches.

Path	N	Average	Minimum	Maximum
Male to son	228	6.48	1.63	23.24
Male to daughter	465	6.26	0.84	23.86
Female to son	193	5.16	0.92	15.76
Female to daughter	408	5.04	0.83	18.22

Approximately 97% of the total Great-billed Seed-Finches analyzed in the reference population had known parents (Table 3). The mean of equivalent complete generations was 4.19. About 60% of birds had five or more known generations in pedigree. For some birds, up to 13 generations of their pedigree have been known.

**Table 3:** Summary of the parameters obtained from pedigree analysis of captive Great-billed Seed-Finches.

Item	Current population (2008 to 2019)
Number of birds	3,939
Number of founders	667
Number of birds with both known parents	3,825
Mean equivalent complete generations	4.19
Percentage of inbred individuals	70.47
Average inbreeding, *F*_*IT*_ (%)	8.26
Average inbreeding of the inbred individuals (%)	11.73
Maximum inbreeding coefficient (%)	43.75
*F*_*ST*_ (%)	3.91
*F*_*IS*_ (%)	4.53
Inbreeding effective population size (*NeF*_*i*_)	19.10
Coancestry effective population size (*NeC*_*i*_)	55.60
Effective number of founders (*fe*)	35.10
Effective number of ancestors (*fa*)	25.93
Founder genome equivalents (*fg*)	12.52
*f*_*a*_/*f*_*e*_ ratio	0.74
*f*_*g*_/*f*_*e*_ ratio	0.36
Number of ancestors explaining 50% of the gene pool	10
Number of ancestors explaining 75% of the gene pool	34
1 –GD (%)	3.99
1 –GD* (%)	1.42
GD*–GD (%)	2.57

*F*_*ST*_ = average inbreeding coefficient under random mating; *F*_*IS*_ = fixation index; 1 –GD = genetic diversity lost in the population since the founder generation; 1 –GD* = loss of genetic diversity due to the unequal contributions of founders; GD*–GD = loss of diversity by genetic drift accumulated over nonfounder generations.

#### Inbreeding and F-statistics

**Fig 2:**
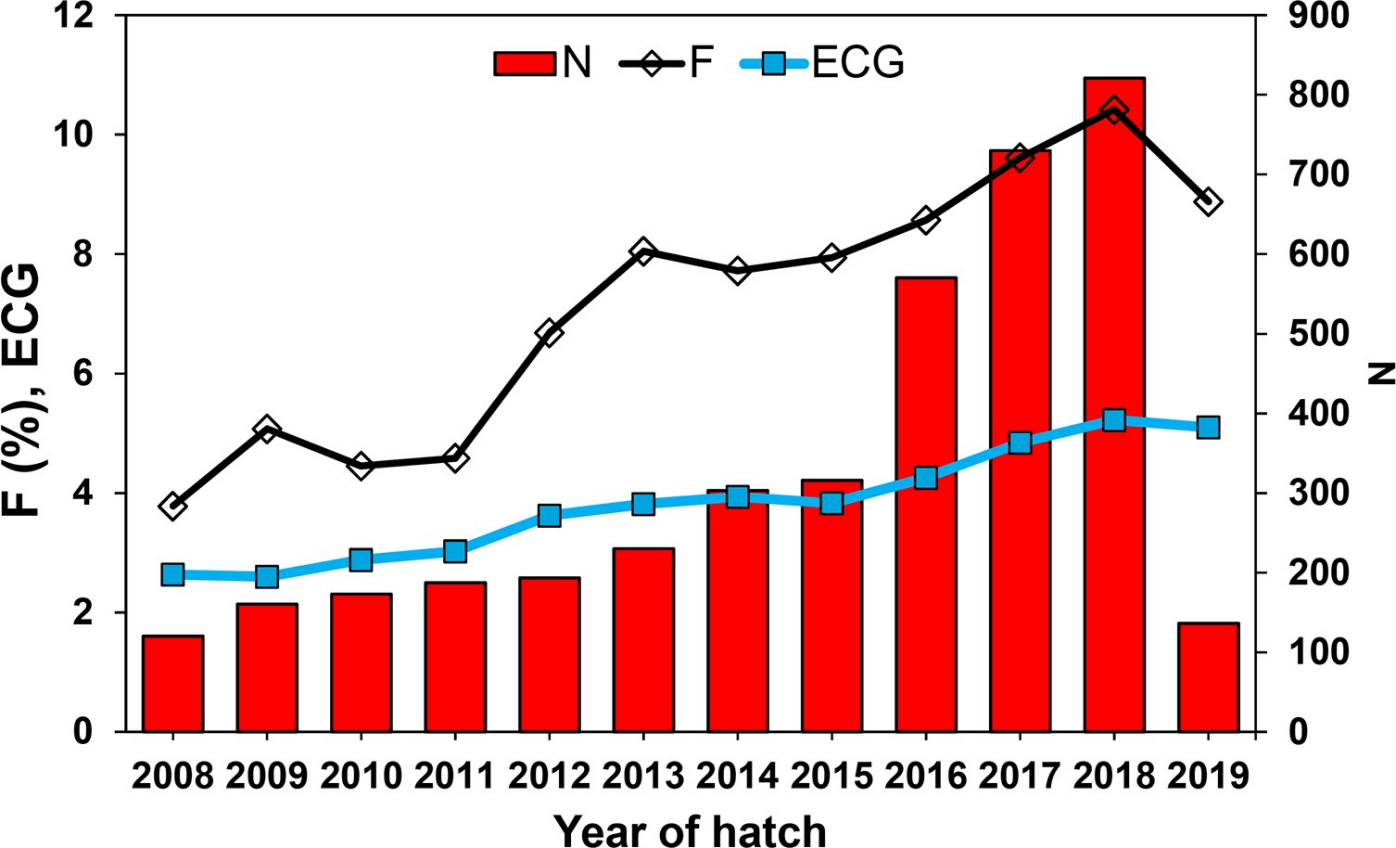
Number of animals born (N), average inbreeding coefficient (*F*), and Equivalent Complete Generations (ECG) according to the year of hatch of captive Great-billed Seed-Finches.

Most birds analyzed were inbred (70.47%). The average inbreeding for the population was 8.26% and reached 11.73% for inbred birds. Some individuals presented an extremely high inbreeding coefficient, above 40%. In the last decade, there has been an increase of 0.57%/year in the average inbreeding of the population (Fig 2). It is important to note that the individual coefficient of inbreeding is sensitive to the quality of the available pedigree information. The observed increase in the inbreeding was accompanied by an increase in mean equivalent complete generations, which indicates greater knowledge of bird pedigree in recent years.

The *F*_*IS*_ value was greater than zero (Table 3), indicating that matings tended to occur among genetically related individuals or that matings occurred preferentially within subpopulations. In this sense, the current coefficient of inbreeding (*F*_*IT*_) was higher than the expected level of inbreeding under random mating (*F*_*ST*_). As an illustration of this scenario, the estimate of effective population size based on coancestry (55.60) was more than double the effective size based on inbreeding rate (19.10). A small effective population size associated with a high level of inbreeding may lead to increased loss of genetic diversity in the next generations.

#### Probabilities of gene origin and genetic diversity

The parameter *f*_*e*_ is dependent on both the number of founders and the balance of their expected contributions to the gene pool. When founders contribute to the reference population more equally, the effective number of founders is higher. In the present population, *f*_*e*_ represented only 5% of the total number of founders (Table 3). In this sense, it could be seen that the founders contributed unbalanced to the current population of captive Great-billed Seed-Finches. The *f*_*e*_ was larger than *f*_*a*_ revealing bottlenecks in the pedigree of the birds, a fact that contributed to the loss of genetic diversity. The parameter *f*_*a*_ complements the information provided by the effective number of founders by accounting for the losses of genetic diversity caused by the unbalanced use of breeding individuals producing bottlenecks. In this sense, only 10 and 34 ancestors explained 50 and 75% of the gene pool of the reference population, respectively. The *f*_*g*_ indicated that less than 13 equally contributing founders would be represented in the current population of captive Great-billed Seed-Finches. The *f*_*g*_/*f*_*e*_ ratio (0.36) supports the loss of genetic diversity due to genetic drift. Approximately 4% of the genetic diversity of the current population was lost, most of which was due to genetic drift.

#### Genetic configuration of subpopulations

**Fig 3:**
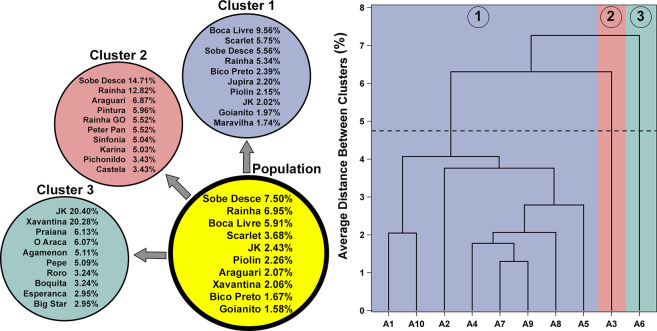
Dendrogram showing the genetic relationships among ten popular commercial aviaries based on Nei's genetic distance (three clusters formed, right). Genetic contribution of the ten most important founders in each cluster and to the population of captive Great-billed Seed-Finches (left) was calculated.

The divergence between the increases in inbreeding and coancestry was reflected by marked differences between estimates of effective population size based on these parameters. The ratio between *NeC*_*i*_ and *NeF*_*i*_ allows to estimate the equivalent number of subpopulations (*S*), which was 2.91 in the present research. Therefore, the current population of captive Great-billed Seed-Finches is subdivided. The dendrogram based on Nei's minimum genetic distance among ten popular and representative Brazilian commercial aviaries (3,880 birds) made it possible to identify subpopulations of captive Great-billed Seed-Finches (Fig 3). Three groups of aviaries were identified considering the overall average genetic distance (4.75%) as a threshold for empirically defining the number of subpopulations. In the first and largest group (1), eight aviaries were grouped, which performed greater or lesser genetic exchange among themselves. Eight of the most important founders of the entire captive Great-billed Seed-Finches population were represented among the top 10 for cluster 1 as Boca Livre (male), Scarlet (female), Sobe Desce (male), Rainha (female), JK (male), Bico Preto (male), Piolin (male) and Goianito (male), for example. Most or even all of these individuals have been known to be wild-caught. These birds are currently important references among breeders of the various lineages of captive Great-billed Seed-Finches. In clusters 2 and 3 were represented the aviaries named 3 and 6, respectively. These aviaries were characterized by genetic isolation and matings almost exclusively among individuals belonging to the subpopulations themselves. The sums of the genetic contributions of the two most important founders for clusters 2 and 3 were 27.53% and 40.68%, respectively.

### Singing time in tournaments and genetic parameters

The average STT was 321.35 seconds (Table 1), indicating that captive Great-billed Seed-Finches sang on average for 35.74% of the time recorded in competitions (900 seconds or 15 minutes). The standard deviation of STT was high and showed a substantial variation in the observed performance of these birds. Some birds reached high STT values, exceeding 12 minutes of singing for a total of 15 minutes. These marks highlight the great singing ability of these birds and also justify the interest of breeders.

**Fig 4:**
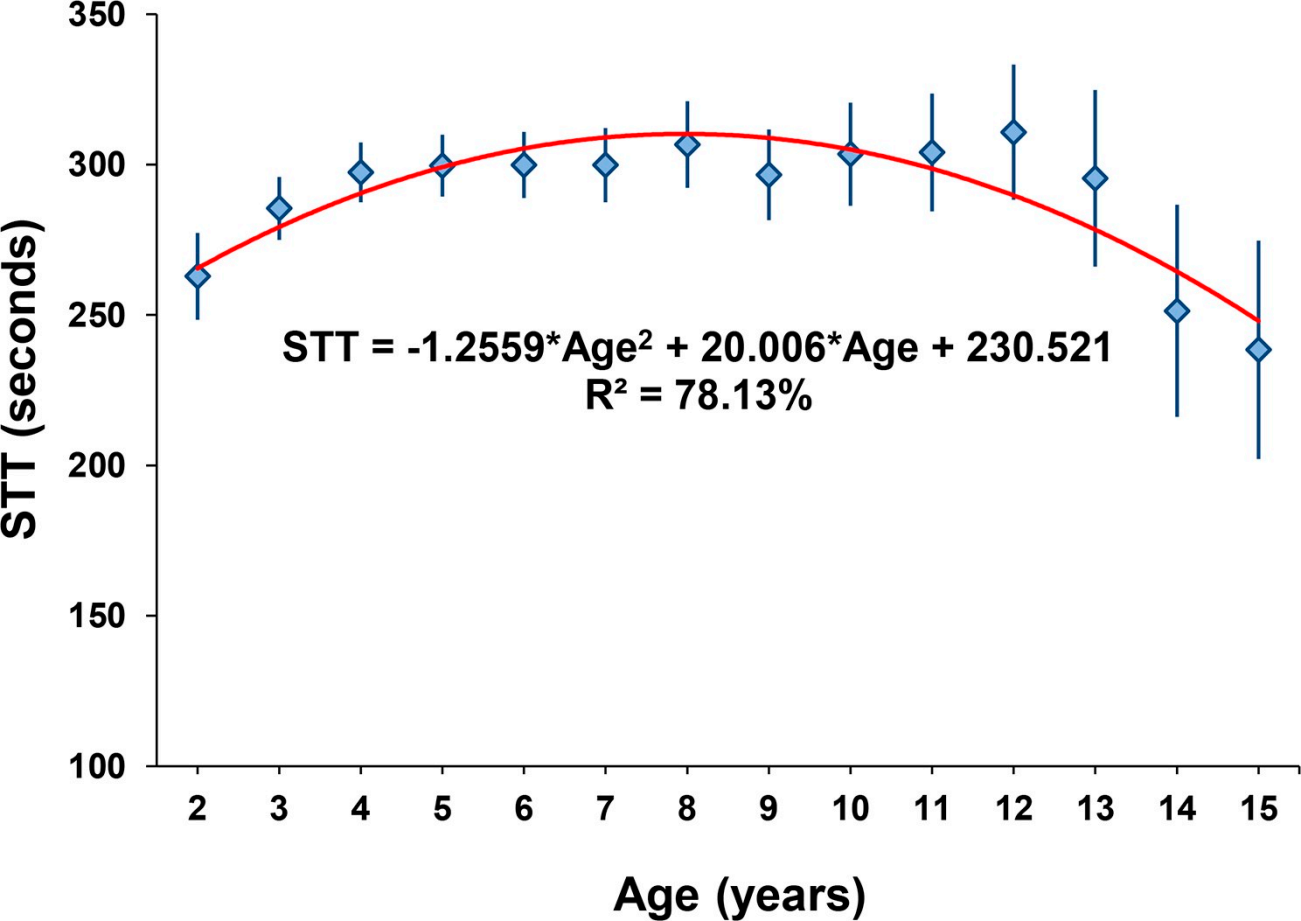
Least square means (diamonds) and respective confidence limits (vertical bars) of singing time in tournaments (STT) according to the age of the bird at the competition. The solid line illustrates the average STT trend obtained by quadratic regression.

The Great-billed Seed-Finches participated in tournaments from young to advanced ages, demonstrating considerable longevity of these animals for competitions. The average age of birds in tournaments was 5.73 years, with the youngest being 1.48 and the oldest 18.75 years old (Table 1). Age influenced (p < 0.001) bird performance for STT (Fig 4) and was therefore included in the model for estimation of genetic parameters. The Great-billed Seed-Finches age at which the STT was maximum was 7.96 years.

Although the sample of animals with STT phenotypes was 602 birds, most of them participated in several events, i.e., they had repeated measurements (average of 12.54 measurements per bird; Table 1). Many of the birds evaluated were genetically related to each other and participated in many of the same events, thus ensuring good connectivity between contemporary groups. The pedigree of birds that were registered for STT included up to six known generations, resulting in a final file of 2,138 animals. The pedigree information used can be considered reliable since Great-billed Seed-Finch breeders regularly perform laboratory paternity tests based on microsatellite analyses. This information made it possible to estimate reasonably accurate genetic parameters, as shown in Table 4.

**Table 4:** Posterior means, Standard Deviations (SD), and highest posterior density intervals (HPD_95%_) for singing time in tournaments of captive Great-billed Seed-Finches.

Parameter	Mean	SD	HPD_95%_
*h*^2^	0.187	0.048	0.099 to 0.290
*I*_*A*_	2.447	0.677	1.266 to 3.947
*c*^2^	0.136	0.040	0.057 to 0.215
*r*	0.323	0.024	0.277 to 0.371
*b*^2^	0.041	0.018	0.011 to 0.081

*h*^2^ = heritability, *I*_*A*_ = evolvability (in %), *c*^2^ = permanent environmental effect as a proportion of the phenotypic variance, *r* = repeatability, *b*^2^ = breeder effect as a proportion of the phenotypic variance.

The narrow-sense heritability estimate for STT was low to moderate, with a posterior mean of 0.187 (0.099 to 0.290; Table 4). Evolvability was 2,447%; thus, STT can respond to selection. Permanent environmental causes represented 13.6% of the phenotypic variance. The posterior mean of repeatability was slightly higher than 0.30, indicating that performance for STT tends to vary over different competitions. The breeder effect corresponded to about 4% of the phenotypic variance of STT.

## Discussion

In the present study, several aspects and parameters, many of which were previously unknown, related to the selection and conservation of captive Great-billed Seed-Finches were revealed. The analysis of a pedigree with more than 6,000 individuals born in the last 30 years made possible several findings. Although molecular techniques can be useful for assessing the genetic diversity, relatedness, and inbreeding of populations even without pedigree information [44,45], they may have limitations when DNA data are incorrectly accessed or based on a small number of informative loci [46,47]. In addition to its low cost, [48] stated that when pedigree knowledge is not limited, pedigree analysis can have some advantages compared to molecular data because whole populations are considered. Thus, these analyses can provide accurate results for a defined generation and time scale. The parameters evaluated here were calculated with reasonable confidence as knowledge of the bird pedigree in this study was moderate, as demonstrated by the mean of equivalent complete generations (4.19).

It has been shown that Great-billed Seed-Finches can be reproductively long-lived, with breeding males and females about 26 and 20 years old, respectively. Thus, a considerable number of birds are expected to exceed 30 years of age, as reported by [49]. With such a long reproductive life, some Great-billed Seed-Finches can have a large amount of progeny [49] reported that a male Great-billed Seed-Finch produced 47 progeny over 18 years. In contrast, our analyses have identified that a single male has produced 185 progeny over more than 20 years. Even so, it is important to note that our estimate may be underestimated since the oldest hatch date of the pedigree analyzed was from 1989. It was also found that the average number of progeny per male and female was relatively small. Great-billed Seed-Finches females are known to lay 2 or 3 eggs, and they can make up to 3 consecutive layings per breeding season [49], especially under captive conditions. Thus, the hypothesis of underestimation of these numbers is not ruled out, as they also depended on spontaneous records of the breeders for the database used in the present study.

Even though kept in the relatively controlled environment of the aviaries in terms of nutrition and management, the breeding period of captive Great-billed Seed-Finches was similar to that observed for free-living individuals between November and March, spring and summer in the south hemisphere [1,50]. Since most breeders keep their birds under natural lighting, photoperiod seems to be one of the major factors determining the breeding of Great-billed Seed-Finches under captivity.

The average generation interval found for the Great-billed captive Seed-finches was relatively high compared with other species of birds such as canary Lizard (*Serinus canaria*) approximately 1.7 years [51] and Stewart Island Robin (*Petroica australis rakiura*) 4 years [23]. Differences in longevity and mating practices explain, at least in part, the observed differences between these species. It is also important to consider that the samples of individuals used in the studies mentioned above were lower than the present study. In terms of selection, long generation intervals may reduce the rate of genetic change. On the other hand, in terms of conservation of genetic diversity, long generation intervals may contribute to preventing the rapid accumulation of inbreeding and genetic drift.

The population of Great-billed Seed-finches captives exhibited a high level of inbreeding (mean of 8.26%) and an alarming trend of increase in the last decade. The cumulative aspect of inbreeding in captive populations is worrisome since, in a few generations, it can lead to fitness impairment [36]. Inbreeding depression has been found for several traits related to the growth, behavior, communication, immune response, reproduction, survival, and song of birds of various species [10–14,52–54]. The level of inbreeding in the population studied may be even higher since this parameter, as calculated here, is dependent on the quality of pedigree information. Pedigrees are known to cover only a limited number of generations, so estimates based on this type of information can be biased downward, as demonstrated by [55]. Analyzing a 23-generation pedigree of Zebra Finch (*Taeniopygia guttata*), those authors estimated an average inbreeding of 4.30% while using an SNP-based technique obtained an estimate of 6.40% (3.6 to 10.20%). With caution in any comparison, the estimate obtained in the present study was within limits observed for other bird populations with an old history of domestication, free-living or even endangered species such as song sparrows (*Melospiza melodia*) 7.60% [47], house sparrows (*Passer domesticus*) 12.9% [52], Lizard canaries (*Serinus canaria*) 15.83% [51], New Zealand hihi (*Notiomystis cincta*) 8.00% [56] and Stewart Island Robin (*Petroica australis rakiura*) approximately 7.00% in the last year evaluated by the author [23]. There is a tendency among breeders to perform mates of close relatives. It is common, for example, to practice mating between parents and their progeny (194 occurrences), or even between siblings (413 occurrences) intended to fix characteristics related to singing and territorialism. Birds originating from these types of mating accounted for 9.75% of all pedigree analyzed. Also, some breeders prefer to perform mating predominantly within specific lineages, notably among descendants of birds that were champions of fiber modality tournaments. In this sense, the current average inbreeding of the population (*F*_*IT*_) was more than twice the inbreeding expected under random mating (*F*_*ST*_). Despite the negative aspects related to effective size and inbreeding depression of restricted matings within subpopulations, the fragmentation into some isolated groups can be beneficial for preserving the genetic diversity of the population. In the long term, as different allelic variants will be fixed in each group, they become genetic reservoirs of variation [8]. The subdivision also serves the interest of breeders to maintain a certain level of differentiation between subpopulations, whether for characteristics related to the song quality, fiber, or morphology.

The values of *f*_*e*_ and *f*_*g*_ were very different from the total number of founders indicating an unbalanced contribution of founders to the current population. The value of *f*_*g*_ was much lower than that of *f*_*e*_, revealing a potential loss of founding alleles in the current population of captive Great-billed Seed-Finches. A similar situation was found by [57] for the highly endangered Takahe (*Porphyrio hochstetteri*) population in New Zealand, where the value of *f*_*g*_ was almost half of the *f*_*e*_. Those authors demonstrated how the unbalanced representation of founders, the high level of inbreeding (8.9%), and the small population size of Takahe contributed to a 7.5% loss of the original heterozygosity. Also, the *f*_*a*_*/f*_*e*_ ratio in the current captive Great-billed Seed-Finches population has evidenced the presence of genetic bottlenecks due to the unbalanced use of birds as breeding animals. The main consequence of pedigree bottlenecks is the loss of genetic diversity, which was about 4% in the present population. Genetic drift was the most important cause of loss of genetic diversity, accounting for about 64% of the total. According to [58], the selection is continually removing some genetic variants and thus reducing total genetic diversity. In small populations or breeding programs where a small number of individuals are used to produce the next generation, genetic variation is mainly lost because of sampling or genetic drift. The loss of genetic diversity in captive and endangered populations should be viewed with concern as it may limit the success of reintroduction projects such as that proposed for Great-billed Seed-Finches by [7]. The goal of many reintroduction programs is to provide sufficient genetic diversity to allow the population to cope with natural selection. In this sense, [23] explained that an adequate abundance of founders is necessary at the beginning of reintroduction to minimize the loss of genetic diversity.

Under non-random mating conditions, coancestry is usually lower than inbreeding, and thus, *F*_*IS*_ assumes a positive value. In the case of captive Great-billed Seed-Finches, this has been verified either by the common practice of mating between relatives, intensive use of some birds as breeding animals, or genetic isolation of some aviaries. The dendrogram based on genetic distances between ten of the most popular commercial aviaries (Fig 3) supported the structuring of the captive Great-billed Seed-Finch population. The representation of founders in the three clusters of aviaries also evidenced the unbalanced genetic contributions of these individuals. As a consequence of population structure, there was a significant discrepancy between estimates of effective population size based on inbreeding (*NeF*_*i*_) and coancestry (*NeC*_*i*_) rates. The *NeF*_*i*_ is more appropriate for cases where the objective is to assess inbreeding depression, which depends on the inbreeding coefficient [30]. However, unlike coancestry, the inbreeding coefficient is greatly influenced by the population structure. Therefore, it is recommended that the effective population size of captive Great-billed Seed-Finches be calculated and evaluated based on coancestry [30,59]. Despite controversies, entities and researchers around the world have reported that a population is at risk of loss of genetic diversity and extinction when the effective population size is below 100 [60], see also discussion in [59]. Captive Great-billed Seed-Finches fall into this situation. There is a consensus that conservation measures should be adopted in such cases.

For the first time, a trait related to singing and territorialism of Great-billed Seed-Finches was defined and evaluated, which was called STT. As stated earlier, Great-billed Seed-Finches are territorial birds [1], which during male-male competition events encourage them to sing. The total time that a bird sings within a predetermined period has been used as the primary criterion for the empirical selection of most captive Great-billed Seed-Finches. Thus, the market and mating practices have been strongly influenced by bird performance for STT. It was revealed during the analysis of phenotypic records of STT that the age of the birds played a quadratic effect on the performance of birds. Thus, it has been shown that around eight years of age, Great-billed Seed-Finches achieve their best performance for STT. It is well known to breeders that birds of this species generally take considerable time to perform well in tournaments compared to their famous congener *Sporophila angolensis*.

The posterior mean of the heritability estimates for STT was of low magnitude (0.187), but with a relatively wide highest posterior density interval (0.099 to 0.290). Thus, it is possible to state that the heritability for STT is statistically different from zero and that this trait can respond to artificial selection. The estimate found here is within limits observed by [21] employing an animal model to analyze various traits related to male Zebra Finches vocalizations, between 0.027 and 0.275. In contrast, using the parent-offspring regression technique to estimate the heritability coefficient, [22] found very variable estimates (0.14 to 1.31) for characteristics related to canary singing. [17] obtained a non-significant heritability estimate for mean or peak song amplitude in Zebra Finch. However, the latter authors pointed out that the statistical power of their analyses was low. Note that heritability estimates are generally lower when obtained from animal models compared to parent-offspring regression. This is due to inflation from other sources of information not properly accounted for by parent-offspring regression [18]. Moreover, according to [18], the animal model produces estimates associated with smaller standard errors. Thus, when possible, the animal model should be preferred for the estimation of more accurate genetic parameters.

Based on the evolvability estimate obtained here, it was possible to predict a 2.45% change for STT per generation under unit selection. Thus, in about four generations, it would be possible to change by about 10% of the STT. Therefore, as stated by [19], a strong directional selection can generate changes in this trait in a relatively short time. These findings can be explained, at least in part, for two reasons: First, the interest of breeders to select STT is relatively recent, the late 1990s and the early 2000s. Second, as the selection was empirically based on individual phenotypic information and tournament awards, this trait was subjected to slight selection pressure in the domestic environment. As a result, the original STT variability in the captive Great-billed Seed-Finches population must be almost perfectly preserved. The high phenotypic standard deviation of STT could confirm this assumption. The heritability, selection intensity, and phenotypic variability associated with a character are factors that determine the response to differential selection [20]. Thus, traits with higher variability tend to exhibit higher selection responses. In this context, [20] stated that the selection intensity applied to a character depends on the organism's reproductive rate, population size, and inbreeding. As previously revealed, the average reproductive rate (probably underestimated) was relatively low in the present population. Still, inbreeding is a concrete problem for captive Great-billed Seed-Finches, and that can impair the reproductive performance of birds. As genetic progress per unit of time is usually more important to be evaluated in a breeding program, the response to selection should also take into account the generation interval. In the bird population studied here, the average generation range was relatively high (5.74 years). Therefore, high inbreeding and generation interval appears to be the main factors that may limit the STT genetic progress of captive Great-billed Seed-Finches.

The repeatability of STT was low but statistically different from zero, as verified by the highest density interval obtained (0.277 to 0.371). Thus, it is expected that many birds with a high STT value in one event will not perform as well in another event and vice versa. It is a common sense of breeders that birds that exhibit stable performance between different competitions are relatively rare. The repeatability estimate for STT was within the range reported in [22], between 0.35 and 0.60 for traits related to canary singing and below the average value (0.67) found for various Zebra Finch male vocal characteristics [21]. As previously reported, the mating of Great-billed Seed-finches is a crucial factor in stimulating male-male competition during tournaments. The vast majority of males are kept side by side with their mates under visual contact before tournaments. In this sense, the female effect on STT should represent an important component of the permanent environment effect.

Finally, it is worth making some additional considerations. Great-billed Seed-Finches have been the subject of concern by researchers, entities, national and international organizations. As stated by [7] and reinforced here, unfortunately, the official environmental organization of Brazil (IBAMA) concentrates action almost exclusively on controlling bird breeders, restricting their actions. This entity is not concerned with the reintroduction of endangered species, much less with the management of the genetic diversity of the captive population and orientation of breeders towards more sustainable breeding. There are currently few concrete initiatives for preserving Great-billed Seed-Finches, such as reintroduction projects [7]. Additionally, the proposed conservation initiatives are restricted to free-living populations. Therefore, as long as captive Great-billed Seed-Finches remain marginalized by authorities and organizations, this population is potentially at risk of extinction. Throughout the domestication history of these birds, there has been no assistance to Great-billed Seed-Finch breeders in Brazil to guide mating, selection, and management of genetic diversity. In recent years, with the popularization of parentage tests based on few microsatellite markers, some breeders have wrongly relied on this information to empirically extrapolate an association with characteristics related to singing and territorialism. This practice has only contributed to increased inbreeding in the population in a dangerous way, as some breeders intentionally mate among more genetically similar individuals. Recently, at the Federal University of Mato Grosso, a public institution in Brazil, the database used in this study was created, which is composed of performance and pedigree information from captive Great-billed Seed-Finches. The project was created to support breeders in order to improve STT genetically, however, without raising the level of inbreeding by applying optimal genetic contribution selection [61]. Selection for STT may contribute to gradually increasing interest in genetically improved captive birds and, consequently, discouraging the capture of the few remaining free-living individuals. Despite the efforts mentioned, it is still very little given the large population of captive Great-billed Seed-Finches and a large number of breeders spread across a country of greater territorial extension. Full cooperation of bird breeders, governmental and non-governmental entities, and universities, as indicated by [7] and also organizations related to the pet sector, should contribute positively to the conservation of genetic diversity and sustainable selection of Great-billed Seed-finches captives.

## Conclusions

The captive Great-billed Seed-Finches population faces many of the problems commonly encountered in domestic animal populations such as a high level of inbreeding, pedigree bottlenecks, unbalanced contribution of breeding animals and structuring. Despite this, most genetic diversity remains preserved within aviaries. The high generation interval found for this population should help to prevent a rapid increase in inbreeding and genetic drift. These results should serve as strong motivation and support for urgent actions to manage the genetic diversity of captive Great-billed Seed-Finches. Greater genetic exchange among aviaries and mating of unrelated individuals are simple measures that can be recommended. From the viewpoint of genetic improvement for singing and territorialism, defined here as STT, this trait presents sufficient variability to allow response to artificial selection. In contrast, inbreeding and high generation interval appear to be the most considerable barriers that may limit the genetic gain for STT in captive Great-billed Seed-Finches. Widespread adoption of techniques such as optimal genetic contribution selection and implementation of routine genetic diversity monitoring via pedigree analysis and molecular tools can be crucial both in terms of breeding and conservation of genetic diversity of captive Great-billed Seed-Finches.

## Supporting information

S1 Data Posterior samples produced in the analysis of variance component estimation. (ZIP)

S2 Data The entire pedigree analyzed in this study. (ZIP)
